# *Aspergillus fumigatus* and Its Allergenic Ribotoxin *Asp f* I: Old Enemies but New Opportunities for Urine-Based Detection of Invasive Pulmonary Aspergillosis Using Lateral-Flow Technology

**DOI:** 10.3390/jof7010019

**Published:** 2020-12-31

**Authors:** Genna Davies, Oski Singh, Juergen Prattes, Martin Hoenigl, Paul W. Sheppard, Christopher R. Thornton

**Affiliations:** 1 ISCA Diagnostics Ltd., Hatherly Laboratories, Prince of Wales Road, Exeter EX4 4PS, UK; g.davies@exeter.ac.uk; 2 Biosciences and MRC Centre for Medical Mycology, Hatherly Laboratories, University of Exeter, Prince of Wales Road, Exeter EX4 4PS, UK; os286@exeter.ac.uk; 3 Department of Internal Medicine, Section of Infectious Diseases and Tropical Medicine, Medical University of Graz, A-8036 Graz, Austria; juergen.prattes@medunigraz.at (J.P.); mhoenigl@health.ucsd.edu (M.H.); 4 Division of Infectious Diseases and Global Public Health, University of California San Diego, San Diego, CA 92093, USA; 5 Vacye Associates, Lowerdown Cottage, Lowerdown, Bovey Tracey TQ13 9LF, UK; paul.sheppard@vacye.com

**Keywords:** monoclonal antibody, ribotoxin, *Asp f* I, *Aspergillus fumigatus*, lateral-flow device, urine

## Abstract

Invasive pulmonary aspergillosis (IPA) caused by *Aspergillus fumigatus* is a life-threatening lung disease of immunocompromised patients. Diagnosis currently relies on non-specific chest CT, culture of the fungus from invasive lung biopsy, and detection of the cell wall carbohydrate galactomannan (GM) in serum or in BAL fluids recovered during invasive bronchoscopy. Urine provides an ideal bodily fluid for the non-invasive detection of pathogen biomarkers, with current urine-based immunodiagnostics for IPA focused on GM. Surrogate protein biomarkers might serve to improve disease detection. Here, we report the development of a monoclonal antibody (mAb), PD7, which is specific to *A. fumigatus* and related species in the section *Fumigati*, and which binds to its 18 kDa ribotoxin *Asp f* I. Using PD7, we show that the protein is secreted during hyphal development, and so represents an ideal candidate for detecting invasive growth. We have developed a lateral-flow device (*Afu*-LFD^®^) incorporating the mAb which has a limit of detection of ~15 ng *Asp f* I/mL urine. Preliminary evidence of the test’s diagnostic potential is demonstrated with urine from a patient with acute lymphoid leukaemia with probable IPA. The *Afu*-LFD^®^ therefore provides a potential novel opportunity for non-invasive urine-based detection of IPA caused by *A. fumigatus*.

## 1. Introduction

*Aspergillus fumigatus* is the most important opportunistic mould pathogen of humans, causing a number of different respiratory diseases including aspergilloma, allergic bronchopulmonary aspergillosis (ABPA), chronic pulmonary aspergillosis (CPA), and invasive pulmonary aspergillosis (IPA), a lethal lung disease of mainly immunocompromised individuals, especially those with prolonged neutropenia, haematological malignancies, and bone marrow and solid organ transplants [1,2]. Recently, IPA has also been identified as a complication of influenza known as Influenza-Associated Pulmonary Aspergillosis (IAPA), and as a life-threatening co-infection of critically ill COVID-19 ICU patients known as COVID-19-Associated Pulmonary Aspergillosis (CAPA) [3,4,5,6,7]. The estimated incidence of IPA is >300,000 cases/year, with more than 90% of the ~150,000 deaths annually caused by *A. fumigatus* [8]. Detection of IPA is notoriously difficult due to non-specific symptoms, and so detection relies on a number of characteristics (host factors, clinical features, and mycological evidence) being present to allow ‘probable’ or ‘proven’ diagnosis according to consensus definitions of invasive fungal diseases [9]. Mycological evidence is reliant on slow and insensitive culture of the pathogen from invasive lung biopsy, or detection of biomarkers in serum [10], and/or in bronchoalveolar lavage fluids (BALf) recovered during invasive bronchoscopy [11]. High-resolution computed tomography (HRCT) of the chest can be used for non-invasive visualisation of lung diseases, but abnormalities in a chest-CT are not pathognomonic of IPA, and so can only be used to raise the suspicion of the disease in high-risk patients [12]. While molecular imaging using antibody-guided positron emission tomography (immunoPET) holds enormous promise for the specific and non-invasive detection of *Aspergillus* lung infections *in vivo* [12,13,14], the technology is in its infancy and relies on sophisticated and expensive technology available in a limited number of hospitals only. Consequently, there is a pressing need for the development of diagnostic tests that allow the detection of *A. fumigatus* biomarkers which are signatures of active infection, which are present in bodily fluids other than serum and invasive BALf, and which are readily accessible in critically ill patients.

Urine is an ideal bodily fluid for the non-invasive detection of pathogen-specific biomarkers. The human urinary proteome contains more than 1500 proteins [15], and changes in urine protein components and concentrations can signify the development of diseases detectable via the transmission of antigens from blood into urine through glomerular filtration of plasma [16]. Based on this principle, a number of immunoassays have been developed for the urine-based detection of bacterial [17], parasitic [18,19,20], and fungal infections [21,22]. Recently, a lateral-flow assay (LFA) was reported that detects fragments of *Aspergillus* carbohydrate in the urine of IPA patients [23]. However, this test and other LFA and ELISA tests for the disease [2] rely on the detection of the same polysaccharide biomarker, the cell wall carbohydrate galactomannan (GM). Detection of *Aspergillus* antigens in urine other than GM might serve to improve IPA diagnosis by allowing a combination biomarker approach to disease detection.

Alternative antigen biomarkers of IPA in urine have been reported previously [1,2], and include an 18 kDa protein secreted by *A. fumigatus* during active growth [24,25,26]. This protein is a ribotoxin, which shares a high degree of sequence and structural homology with the dimeric 36 kDa ribotoxin mitogillin from *A. fumigatus* [27], the 18 kDa ribotoxin restrictocin from *Aspergillus restrictus* [28], the ribotoxin α-sarcin from *Aspergillus giganteus* [29,30], and the ribotoxin c-sarcin (clavin) from *Aspergillus clavatus* [31]. Furthermore, it has been shown that the 18 kDa ribotoxin is synonymous with the *A. fumigatus* ribotoxin *Asp f* I [32,33], a major IgE-binding allergen implicated in ABPA, aspergilloma, and cystic fibrosis with complication of ABPA [34,35,36,37,38,39].

In this paper, we report the development of a murine IgG1 mAb, PD7^®^, raised against the *A. fumigatus* ribotoxin mitogillin, and which binds to an epitope conserved amongst the *Aspergillus* ribotoxins mitogillin, *Asp f* I, restrictocin, and α-sarcin. Using the mAb, which is specific to *A. fumigatus* and clinically-relevant sibling species in the section *Fumigati* [40,41,42], we have developed a lateral-flow assay known as *Afu*-LFD^®^ (*Aspergillus fumigatus*-Lateral Flow Device) and a sandwich ELISA (*Afu*-ELISA^®^) for the detection of the protein biomarker. Furthermore, using the *Afu*-LFD^®^ immunoassay, we have developed a rapid and non-invasive urine-based test for IPA in humans caused by *A. fumigatus*.

## 2. Materials and Methods

### 2.1. Ethics Statement

Hybridoma work described in this study was conducted under a UK Home Office Project License, and was reviewed by the institution’s Animal Welfare Ethical Review Board (AWERB) for approval. The work was carried out in accordance with The Animals (Scientific Procedures) Act 1986 Directive 2010/63/EU, and followed all the Codes of Practice which reinforce this law, including all elements of housing, care, and euthanasia of the animals. For collection of human urine samples, the study adhered to the Declaration of Helsinki, 2013, Good Clinical Practice, and was approved by the local ethics committee, Medical University of Graz, Austria (EC number 23-343).

### 2.2. Preparation of Immunogen and Immunisation Regime

For hybridoma production, mice were immunised with recombinant mitogillin (mitF; MyBioSource, MBS1189059). The immunogen comprising mitF diluted to 1mg protein/mL buffer was stored at −20 °C before animal immunisations. For immunisations, 6-wk-old BALB/c white mice were each given four intra-peritoneal injections (300 µL per injection) of immunogen at 2 wk intervals, and a single booster injection was given five days before fusion.

### 2.3. Production of Hybridomas and Screening by PTA-ELISA

Hybridoma cells were produced by the method described elsewhere [43], and monoclonal antibody (mAb)-producing clones identified in Plate-Trapped Antigen Enzyme-Linked Immunosorbent Assay (PTA-ELISA) tests, by using the immunogen diluted in phosphate-buffered saline (PBS; 137 mM NaCl, 2.7 mM KCl, 8 mM Na_2_HPO_4_, 1.5 mM KH_2_PO_4_ [pH 7.2]) and immobilised to Maxisorp microtiter plate wells (Nunc) at 50 µL/well. Wells containing immobilised antigen were incubated with 50 µL of mAb hybridoma tissue culture supernatant (TCS) for 1 h, after which wells were washed three times, for 5 min each, with PBST (PBS containing 0.05% (vol:vol) Tween-20). Goat anti-mouse polyvalent immunoglobulin (G, A, M) peroxidase conjugate (A0412, Sigma, St. Louis, MI, USA), diluted 1:1000 in PBST, was added to the wells and incubated for a further hour. The plates were washed with PBST as described, given a final 5 min wash with PBS, and bound antibody visualised by incubating wells with tetramethyl benzidine (TMB) substrate solution for 30 min, after which reactions were stopped by the addition of 3 M H_2_SO_4_. Absorbance values were determined at 450 nm using a microplate reader (Tecan GENios, Tecan Austria GmbH, Grödig, Austria). Control wells were incubated with tissue culture medium (TCM) containing 10% (vol:vol) foetal bovine serum (FBS; FCS-SA, Labtech International Ltd., Heathfield, UK) only. All incubation steps were performed at 23 °C in sealed plastic bags. The threshold for detection of the antigen in PTA-ELISA was determined from control means (2 × TCM absorbance values). These values were consistently in the range of 0.050–0.100. Consequently, absorbance values ≥ 0.100 were considered as positive for the detection of antigen.

### 2.4. Determination of Ig Class and Sub-Cloning Procedure

The Ig class of mAbs was determined by using PTA-ELISA. Wells of microtiter plates coated with immunogen were incubated successively with hybridoma TCS for 1 h, followed by goat anti-mouse IgG1-, IgG2a-, IgG2b-, IgG3-, IgM- or IgA-specific antiserum (ISO-2, Sigma) diluted 1:3000 in PBST for 30 min, and rabbit anti-goat peroxidase conjugate (A5420, Sigma) diluted 1:1000 for a further 30 min. Bound antibody was visualised with TMB substrate as described. Hybridoma cell lines were sub-cloned three times by limiting dilution, and cell lines were grown in bulk in a non-selective medium, preserved by slowly freezing in FBS/dimethyl sulfoxide (92:8 vol:vol), and stored in liquid N_2_.

### 2.5. Purification and Enzyme Conjugation of mAbs

Hybridoma TCS was harvested by centrifugation at 2147× *g* for 40 min at 4 °C, followed by filtration through a 0.8 μM cellulose acetate filter (10462240, Fisher Scientific, Loughborough, UK). Culture supernatant was loaded onto a HiTrap protein A column (17-0402-01, GE Healthcare Life Sciences) using a peristaltic pump P-1 (18-1110-91, GE Healthcare Life Sciences) with a low pulsation flow of 1 mL/min. Columns were equilibrated with 10 mL PBS, and column-bound antibody was eluted with 5 mL of 0.1 M glycine-HCl buffer (pH 2.5) with a flow rate of 0.5 mL/min. The buffer of the purified antibody was exchanged to PBS using a disposable PD-10 desalting column (17-0851-01, GE Healthcare Life Sciences). Following purification, the antibody was sterile filtered with a 0.24 µm syringe filter (85037-574-44, Fisher Scientific, Loughborough, UK) and stored at 4 °C. Protein concentration was determined using a Nanodrop spectrophotometer with protein concentration calculated using the mass extinction coefficient of 13.7 at 280 nm for a 1% (10mg/mL) IgG solution. Antibody purity was confirmed by SDS-PAGE and gel staining using Coomassie Brilliant Blue R-250 dye (Thermo Fisher Scientific, Waltham, MA, USA). Protein A-purified mAb PD7 or mAb JF5 [12] was conjugated to horseradish peroxidase (PD7-HRP or JF5-HRP) for ELISA studies using a Lightning-Link horseradish peroxidase conjugation kit (701-0000; Bio-Techne Ltd., Abingdon, UK), or to alkaline phosphatase (PD7-AKP or JF5-AKP) for Western blotting studies using a Lightning-Link alkaline phosphatase conjugation kit (702-0010; Bio-Techne Ltd.).

### 2.6. PD7 Recognition of Aspergillus Ribotoxins by SDS-PAGE and Western Blotting

Recognition of different *Aspergillus* ribotoxins by mAb PD7 was determined by sodium-dodecyl-sulphate-polyacrylamide gel electrophoresis (SDS-PAGE) and Western blotting, using 4–20% gradient polyacrylamide gels (4561094, Bio-Rad) under denaturing conditions. Proteins were separated electrophoretically at 165 V, and pre-stained markers (1610377, Bio-Rad) were used for molecular weight determinations. For Western blotting, separated proteins were transferred electrophoretically onto a PVDF membrane (1620175, Bio-Rad) for 2 h at 75 V, and the membrane was blocked for 16 h at 4 °C in PBS containing 1% (wt:vol) BSA. Blocked membranes were incubated with PD7-AKP conjugate diluted 1 in 5000 in PBS containing 0.5% (wt:vol) BSA (PBSA) for 2 h at 23 °C. Membranes were washed three times with PBS, once with PBST, and bound antibody visualised by incubation in substrate solution [43]. Reactions were stopped by immersing membranes in dH_2_O, and membranes were then air-dried between sheets of Whatman filter paper.

### 2.7. Binding Kinetics of mAb PD7 and Limits of Detection in PTA-ELISA

Binding kinetics of mAb PD7 were measured using Bio-Layer Interferometry (BLI) technology from ForteBio (using the BLItz biosensor system, ForteBio, Pall). For all steps, sterile filtered PBS was used as running buffer, and solutions were agitated at 1000 RPM. Following an initial baseline of 30 s, protein A-purified PD7, at a concentration of 50 µg/mL, was immobilised on anti-mouse Fc capture biosensors (ForteBio, 18-5088) for 120 s. After a second baseline step of 60 s, bound PD7 was associated with recombinant mitF, between 69.44 nM and 277.8 nM, for 60 s. This was followed by dissociation in PBS for 120 s. A reference, PD7-bound, biosensor with association and dissociation steps in PBS alone was used for background correction. ForteBio analysis software was used to fit binding curves to a global 1:1 binding model and to calculate kinetic constants.

### 2.8. Heat Stability, Native PAGE and Western Blotting

Heat stability of the PD7 epitope was determined by heating mitF and *Asp f* I proteins in a boiling water bath. At 10 min intervals, 50 μL volumes were removed and, after cooling, were transferred to the wells of microtitre wells for assay by ELISA using mAb PD7. For native PAGE, samples were heated for different time periods, diluted in native sample buffer (1610738, Bio-Rad), and then electrophoresed under non-denaturing conditions using 4–20% gradient polyacrylamide gels (4561094, Bio-Rad), prior to Western blotting, and processing with mAb PD7, as described.

### 2.9. Fungal Culture

Fungi (Table 1) were routinely cultured on Malt Extract Agar (MEA; 20 g each of malt extract broth (MEB; LP0039, Thermo Fisher Scientific Oxoid Ltd., Basingstoke, UK and agar No. 2 (Neogen) in 1 L distilled water). The medium was autoclaved at 121 °C for 15 min prior to use, and fungi were grown at 37 °C under a 16 h fluorescent light regime. For recovery of *A. fumigatus* strains from naturally-infected soils, samples were sprinkled on the surface of MEA containing 50 mg/L chloramphenicol, and then incubated at 37 °C for 48 h. Putative isolates of *A. fumigatus*, identified by gross morphology and microscopy, were single-spore isolated and then grown in axenic culture on MEA. Polymerase chain reaction was then used to confirm identity using *A. fumigatus*-specific primers which amplify the *Asp f* I-encoding gene [44].

### 2.10. Generation of A. fumigatus Ribotoxin-Deficient Mutants

Targeted gene replacement of the *A. fumigatus* mitogillin-encoding gene (*mitF*) with the hygromycin B phosphotransferase-encoding gene (*hph*) was performed using the split marker recombination strategy as described previously [13,47,48]. The *mitF* gene and flanking sequences were obtained from the *Aspergillus* Genome Database (AspGD, http://www.aspergillusgenome.org/), and used to design primers accordingly (Table S1). Primer pairs mitF_LF.1_F/mitF_LF.1_R and mitF_RF.1_F/mitF_RF.1_R were used to amplify the 5′ (LF, 0.8 kb) and 3′ (RF, 1.0 kb) flanking regions of the *mitF* gene, respectively, from *A. fumigatus* Af293 genomic DNA. Simultaneously, split *hph* templates were amplified to create the 5′ amplicon (HY, 1.2 kb) using primers HY split/M13F, and 3′ amplicon (YG, 0.8 kb) using primers YG split/M13R. Fusion PCR resulted in two products, LFHY (2.0 kb) and RFYG (1.8 kb) using primer pairs mitF_LF.1_F/HY split and mitF_RF.1_R/YG split, respectively. The amplicons were gel purified and used in Af293 protoplast transformation giving way to replacement of *mitF* with the successfully assembled *hph* gene, conferring hygromycin B resistance. Putative ΔAf*mitF*::*hph* transformants were selected in the presence of hygromycin B (600 µg/mL) and gene replacement was confirmed by *size difference* PCR. PCR products were amplified from genomic DNA using the primer pair mitF_LF.1_F/mitF_RF.1_R. The products therefore contained both the LF (0.8 kb) and RF (1.0 kb) flanking regions of the *mitF* gene, with either the *mitF* (0.5 kb) or assembled *hph* gene (1.6 kb), with predicted sizes of 2.5 kb for the wild-type strain Af293 or 3.6 kb for gene replacement mutants.

### 2.11. Production of Ribotoxins In Vitro

For ribotoxin production studies, *Aspergillus* species were grown in liquid *Aspergillus* Minimal Medium (AMM) [49]. Unrelated species were grown in MEB, but were otherwise treated similarly. Three replicate 250 mL conical flasks containing 100 mL of autoclaved medium were inoculated with spores to a final concentration of 10^3^ spores/mL, and the cultures incubated at 37 °C with shaking (120 RPM) in a New Brunswick orbital shaker. For the *A. fumigatus* sporulation-deficient mutant ΔAf*brlA*, the flasks were inoculated with 3 mm × 5 mm plugs of mycelium taken from the leading edge of a culture grown for 48 h at 37 °C on MEB. At 24 h intervals, replicate flasks were harvested and culture fluids separated from mycelium by filtration through Miracloth. Mycelial biomass was dried for 4 d at 80 °C and weighed. Culture filtrates were mixed with ethanol at a ratio of 1:4 (vol:vol) and chilled at −20 °C for 16 h to allow protein precipitation. After centrifugation at 4 °C for 10 min at 3202× *g*, protein pellets were washed once with chilled ethanol, and centrifuged for a further 5 min. The clear supernatants were aspirated, the pellets air-dried, re-suspended in PBS, and protein suspensions stored at −80 °C. On thawing, any insoluble material was removed by centrifugation for 5 min at 16,000× *g*, and solutions heated for 10 min in a boiling water bath prior to immunoassay by *Afu*-ELISA^®^, or SDS-PAGE and Western blotting. The heating step was not required for assay by PTA-ELISA.

For colony blots, *A. fumigatus* Af293 and the ribotoxin-deficient ΔAf*mitF*::*hph* mutants were grown for 48 h at 37 °C on MEB, after which time the colonies were overlayed with PVDF membrane for 8 h. The membranes were blocked and processed with PD7-AKP or JF5-AKP conjugates as described for Western blotting.

### 2.12. Afu-ELISA^®^

Wells of Maxisorp microtiter plate wells (Nunc) were coated for 16 h at 4 °C with 50 μL volumes of protein A-purified mAb PD7 at a concentration of 1 µg/mL in PBS. The wells were incubated with heat-treated protein solutions for 2 h, washed three times (5 min each) with PBST, and then incubated for 1 h with PD7-HRP conjugate diluted 1 in 1000 in PBST (equivalent to ~1 μg antibody protein/mL buffer). The wells were washed with PBST as described, given a final 5 min wash with PBS, and bound antibody visualised by incubating wells with tetramethyl benzidine (TMB) substrate solution for 30 min, after which reactions were stopped by the addition of 3 M H_2_SO_4_. Absorbance values were determined at 450 nm using a microplate reader (Tecan GENios, Tecan Austria GmbH). All incubation steps were performed at 23 °C in sealed plastic bags. The threshold for detection of the PD7 antigen in *Afu*-ELISA^®^ was determined from the means of controls (AMM or MEB only). These values were consistently in the range of 0.050–0.100. Consequently, absorbance values ≥ 0.100 were considered as positive for the detection of antigen. The limit of detection of the *Afu*-ELISA^®^ was determined from a calibration curve of known concentrations of heat-treated *Asp f* I diluted in PBST.

### 2.13. Detection of Asp f I in Urine

#### 2.13.1. Configuration of the Lateral-Flow Device

The lateral-flow assay, known as *Afu*-LFD^®^, was manufactured by Lateral Dx (Alloa, Scotland, UK). The test consisted of Kenosha 75 mm backing card; Ahlstrom 222 and 1281 top and sample pads, respectively; and a Sartorius CN95 nitrocellulose membrane. Monoclonal antibody PD7 was conjugated to NanoAct^®^ Red CNB particles (Asahi Kasei) according to the manufacturer’s instructions, and applied to the release pad. The test line antibody consisted of mAb PD7 at 1 mg protein/mL, while a commercial goat anti-mouse IgG (BBI Solutions) acted as the control line.

#### 2.13.2. Limit of Detection of the Afu-LFD^®^ Test

The limit of detection (LOD) of the *Afu*-LFD^®^ test was determined using first void urine (FVU) samples from three independent healthy donors. Five hundred μL volumes of urine spiked with known concentrations of recombinant *Asp f* I were added to Amicon Ultra-0.5 centrifugal (10 kDa cut-off) filter units (Sigma, UFC5010BK), and concentrated 10-fold by centrifugation for 5 min at 14,000× *g*, followed by buffer exchange with 400 μL PBS for 8 min at 14,000× *g*. Concentrated samples were recovered by centrifugation at 1000× *g* for 2 min, and then heated for 10 min in a boiling water bath. Cooled samples were then mixed 1:1 (vol:vol) with 2× PBS containing 0.2% (vol:vol) Tween-20, and the resultant 100 μL volumes then added to *Afu*-LFD^®^ devices. The negative control consisted of un-spiked FVU, which was otherwise processed according to spiked samples. Test results were recorded after 30 min as negative [single internal control (C) line only] or positive [both control (C) and test (T) lines visible] for the PD7 protein biomarker.

### 2.14. Processing and Testing of Patient Urine Samples Using the Afu-LFD^®^ Test

Patient urine samples stored at −80 °C were thawed, vortexed briefly, and then centrifuged for 10 min at 16,000× *g*. Five hundred μL of urine supernatant was added to an Amicon Ultra-0.5 centrifugal (10 kDa cut-off) filter unit (Sigma, UFC5010BK), and concentrated 10-fold by centrifugation for 5 min at 14,000× *g*, followed by buffer exchange with 400 μL PBS for 8 min at 14,000× *g*. The concentrated sample was recovered by centrifugation at 1000× *g* for 2 min, and then heated for 10 min in a boiling water bath. For *Afu*-LFD^®^ tests, the cooled sample was mixed 1:1 (vol:vol) with 2× PBS containing 0.2% (vol:vol) Tween-20, and the resultant 100 μL volume then added to an *Afu*-LFD^®^ device. Test results were recorded after 30 min as negative [single internal control (C) line only] or positive [both control (C) and test (T) lines visible] for the PD7 protein biomarker.

### 2.15. SDS-PAGE, Western Blotting, and LC–MS of Patient Urine Sample

For SDS-PAGE and Western blotting, concentrated and buffered-exchanged urine samples were mixed with Laemmli buffer, and processed with mAb PD7 as described previously. Immuno-reactive bands were located on replica SDS-PAGE gels stained with Coomassie Blue, removed with a scalpel blade and digested with trypsin using a ProGest automated digestion unit (Perkin Elmer Life Sciences (UK) Ltd., Beaconsfield, UK). The resulting peptides were analysed by mass spectrometry using a 4700 MALDI-TofTof mass spectrometer (Applied Biosystems, Foster City, CA, USA) to give a peptide mass fingerprint and peptide sequence information, which was searched against various databases using the Mascot search programme (www.matrixscience.com) to identify the protein present in the gel band.

### 2.16. Statistical Analysis

All statistical analyses were performed using RStudio software with the Agricolae extension package. ANOVA was conducted with null hypothesis. Where *p* < 0.05, the null hypothesis was rejected and a post-hoc Tukey test was conducted.

## 3. Results

### 3.1. Production of Hybridomas and mAb Isotyping

Two hybridoma fusions were performed, and 855 hybridoma cell lines were tested in PTA-ELISA for recognition of the immunogen. Thirty-six cell lines produced immuno-reactive antibodies, with 21 producing mAbs of the immunoglobulin class G. The cell line PD7 was selected for further testing based on the strength of its reaction with the immunogen in ELISA, and its ability to recognize native protein produced by *A. fumigatus* Af293. Isotyping of mAb PD7 showed that it belonged to immunoglobulin class G1 (IgG1).

### 3.2. Recognition of Aspergillus Ribotoxins by mAb PD7

**Figure 1:**
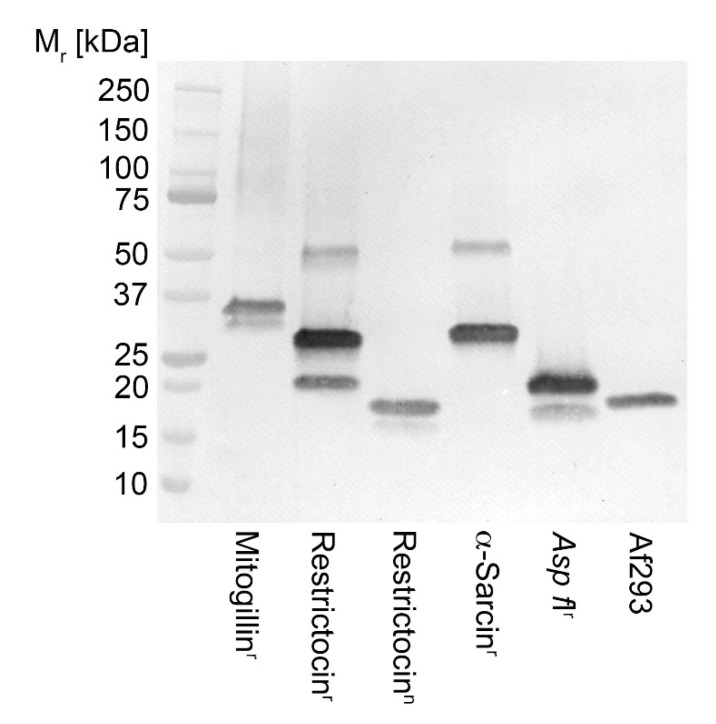
Western immunoblot of different *Aspergillus* ribotoxins using mAb PD7. Mitogillin^r^ = recombinant mitogillin (mitF) from *Aspergillus fumigatus* (MBS1189059); Restrictocin^r^ = recombinant restrictocin from *Aspergillus restrictus* (MBS1228220); Restrictocin^n^ = native restrictocin from *Aspergillus restrictus* (Sigma R0381); α-Sarcin^r^ = recombinant α-sarcin from *Aspergillus giganteus* (MBS1239731); *Asp f* I^r^ = recombinant *Asp f* I from *A. fumigatus* (RP-AF1-1); Af293 = protein precipitate from 72-h-old AMM culture filtrate of *Aspergillus fumigatus* Af293 (this study). Each well contains 40 ng of protein.

In SDS-PAGE and Western blotting studies, mAb PD7 bound to all six of the *Aspergillus* ribotoxins tested (Figure 1). Binding of PD7 to the immunogen (recombinant mitogillin) is in keeping with the estimated molecular weight for this protein dimer (~36 kDa). Native restrictocin, and protein precipitate prepared from 72-h-old AMM culture filtrate of *A. fumigatus* Af293 (this study), yielded PD7-reactive bands of ~18 kDa, consistent with the monomeric *Asp f* I ribotoxin produced by the pathogen. A recombinant form of this *Asp f* I ribotoxin gave a major PD7-reactive band of ~19 kDa, comprising the 18 kDa protein and an additional 1 kDa from a 6xHis-tag. Major PD7-reactive bands of ~30 kDa were present in the recombinant restrictocin and α-sarcin preparations, in addition to immuno-reactive bands with higher (~50 kDa, both preparations) and lower (~19 kDa, recombinant restrictocin only) molecular weights.

### 3.3. Binding Kinetics of mAb PD7 and Limits of Detection of PTA-ELISA and Afu-ELISA^®^

The equilibrium dissociation constant (K_D_) of mAb PD7 determined by Bio-Layer Interferometry (Figure S1A) using the dimer mitF was 1.02 × 10^−7^ (Table S2), showing that mAb PD7 has nM sensitivity. The limit of antigen detection in the PTA-ELISA was determined as 4 ng/mL for both the dimer mitF and for the monomer *Asp f* I (Figure S1B), while the limit of detection of the *Afu*-ELISA^®^ was determined as ~15 ng *Asp f* I/mL (Figure S1C).

### 3.4. Heat Stability of the PD7 Epitope and Protein Aggregation

The epitope bound by mAb PD7 is heat stable, with no significant effect on mAb binding following 60 min heating of the 36 kDa dimer mitogillin (Figure S2A). Binding of PD7 to the monomeric protein *Asp f* I was unaffected by heat treatment for up to 30 min, but there was a significant progressive reduction in mAb binding thereafter (Figure S2A). Under native conditions, heating of *Asp f* I led to progressive increases in PD7 binding in Western blots over time (Figure S2B).

### 3.5. Production of Ribotoxins In Vitro

#### *A. fumigatus* Af293 and Ribotoxin-Deficient Mutants

**Figure 2:**
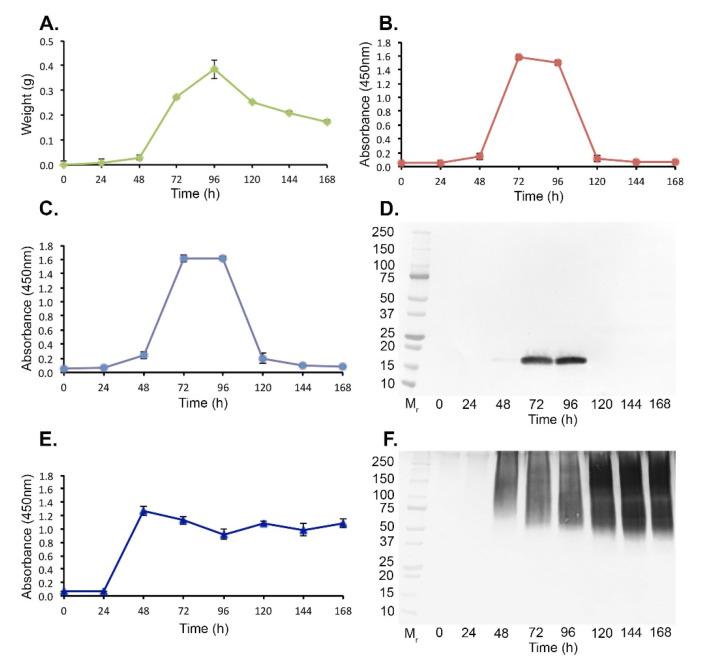
Production of the 18 kDa ribotoxin by *A. fumigatus* Af293 in vitro. (**A**) Dry weights of the pathogen over the 7 day experimental period. (**B**) *Afu*-ELISA^®^ of AMM protein precipitates, with the same samples tested by PTA-ELISA (**C**), using mAb PD7. Each data point in figures (**A**) to (**C**) is the mean of three replicates ±SE, and the threshold absorbance value for detection of protein in the *Afu*-ELISA^®^ is ≥0.100. (**D**) Western blot of pooled replicate AMM protein precipitates using mAb PD7, showing weak detection of an 18 kDa immuno-reactive band at 48 h post-inoculation, strong detection at 72 h and 96 h post-inoculation, and no detection thereafter. (**E**) PTA-ELISA detection of galactofuranose-rich peptidoglycans in protein precipitates using mAb JF5. Each data point is the mean absorbance value of three replicates ±SE, and the threshold absorbance value for detection of glycoprotein in PTA-ELISA is ≥0.100. (**F**) Western blot of pooled AMM protein precipitates using mAb JF5.

Hyphal growth of *A. fumigatus* Af293 in AMM shake culture peaked at 96 h post-inoculation with a mean dry weight of 0.384 g ± 0.037 g (Figure 2A), and coincided with sporulation by the fungus. Ribotoxin production, determined by both *Afu*-ELISA^®^ (Figure 2B) and PTA-ELISA (Figure 2C) using mAb PD7, followed a similar trend, with ribotoxin production first detected 48 h post-inoculation (absorbance values of ≥0.100, the threshold value for antigen positivity in both immunoassays), maximum ribotoxin production detected between 72 h and 96 h post-inoculation, and with a rapid decline after 96 h coincident with the cessation of hyphal growth and onset of sporulation. Western blot analysis (Figure 2D) using representative samples at each time point, revealed a similar trend to ELISA tests, with production of a single 18 kDa ribotoxin first discernible at 48 h post-inoculation, stronger detection at both 72 h and 96 h post-inoculation, and absence of production thereafter. As a means of comparison with other extracellular *Aspergillus* antigens, we tested the same samples by PTA-ELISA (Figure 2E) and Western blot (Figure 2F) with mAb JF5, an *Aspergillus-*specific mAb that binds to galactofuranose-rich peptidoglycans. The dynamics of JF5 antigen production were markedly different to those of ribotoxin production, with antigen production peaking at 48 h post-inoculation and plateauing thereafter. This shows that while both antigens are secreted into the culture filtrate in the early phases of fungal growth, and both require germination of spores for antigen production, ribotoxin production is strictly limited to the hyphal growth phase, and does not accumulate post-sporulation, unlike secreted peptidoglycans.

Mutants of *A. fumigatus* generated by targeted deletion of the ribotoxin-encoding gene *mitF* (Figure S3A) were deficient in ribotoxin production, but otherwise retained similar phenotypic characteristics (biomass accumulation in liquid culture) to those of the wild-type strain Af293 (Figure S3B). Protein precipitates from AMM cultures of two independent mutant strains (ΔAf*mitF*::*hph*2.1 and ΔAf*mitF*::*hph*3.4) both failed to react with mAb PD7 in Western blotting (Figure S3C) tests, whereas they retained activity in Western blots with mAb JF5 (Figure S3D). Colony blots of these strains (Figure S3E) showed reaction of mAb PD7 with the leading edge of Af293 colonies, but lack of reactivity with the mutant strains. As positive controls, blots probed with mAb JF5 showed intense staining of galactofuranose-rich peptidoglycans secreted by the leading edges of both the wild-type and mutant strains (Figure S3E).

### 3.6. Ribotoxin Production by the A. fumigatus Sporulation-Deficient Mutant ΔAfbrlA

**Figure 3:**
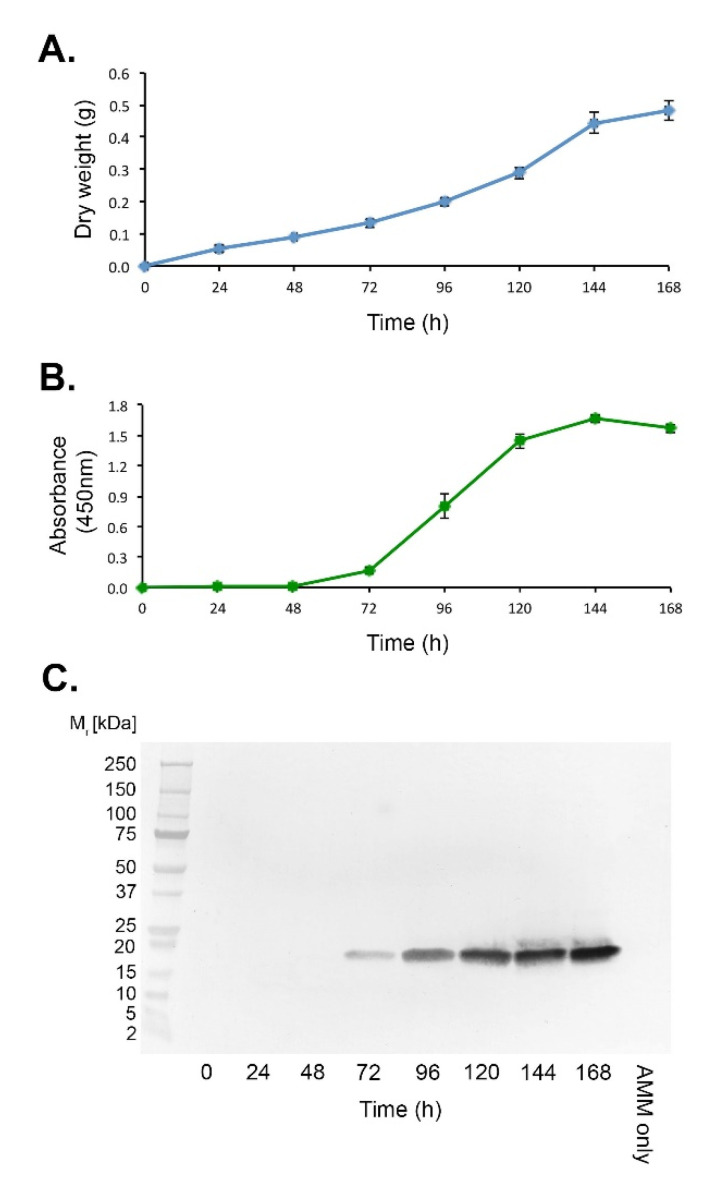
Production of the 18 kDa ribotoxin by the *A. fumigatus* sporulation-deficient mutant ΔAf*brlA in vitro*. (**A**) Dry weights of the mutant over the 7 day experimental period. (**B**) *Afu*-ELISA^®^ of AMM protein precipitates using mAb PD7. Each data point in figures (**A**,**B**) is the mean of three replicates ±SE, and the threshold absorbance value for detection of protein in the *Afu*-ELISA^®^ (**B**) is ≥0.100. (**C**) Western blot of pooled protein samples, showing the appearance of the PD7-reactive 18 kDa protein at 72 h post-inoculation and at each time point thereafter. The negative control comprised AMM only.

Hyphal growth of the sporulation-deficient mutant ΔAf*brlA* in AMM shake culture increased steadily over time and started to plateau at 168 h post-inoculation (Figure 3A). Ribotoxin production, determined by *Afu*-ELISA^®^ using mAb PD7 (Figure 3B), was first detected 72 h post-inoculation, with production increasing steadily thereafter until 144 h. Production then decreased concomitant with the plateauing in biomass accumulation. Western blot analysis (Figure 3C) using representative samples at each time point, revealed a similar trend to the *Afu*-ELISA^®^ test, with production of a single 18 kDa ribotoxin first discernible at 72 h post-inoculation, and with detection at all time points thereafter.

### 3.7. Production of Ribotoxins by Other Aspergillus Fumigatus Strains, Non-Fumigatus Aspergillus Species, and Unrelated Human Pathogenic Fungi

**Figure 4:**
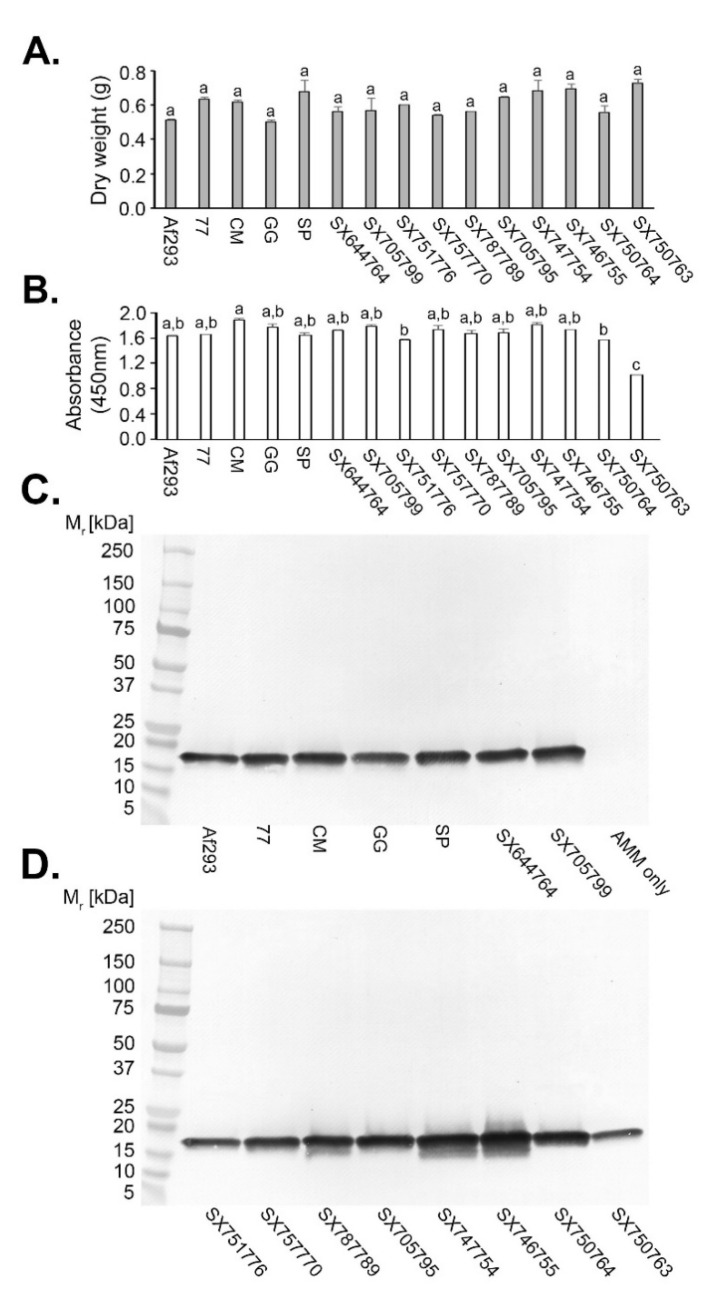
Production of the PD7-reactive 18 kDa ribotoxin by different strains of *A. fumigatus*. All 14 strains were isolated from naturally-infested soils, and protein precipitates were prepared from 72-h-old AMM shake cultures. *A. fumigatus* Af293 acted as the positive control, while AMM only acted as the negative control. (**A**) Dry weights of strains after 72 h growth, showing no significant differences in weights compared to Af293. (**B**) While the mean absorbance value for strain SX750763 was significantly reduced in the *Afu*-ELISA^®^, an 18 kDa PD7-reactive band was present in all protein precipitates (**C**,**D**), with the exception of the negative control. Bars in (**A**,**B**) are the means of three replicate values ±SE, and bars with the same letter are not significantly different at *p* < 0.05. The threshold absorbance value for detection of ribotoxin protein in the *Afu*-ELISA^®^ (**B**) is ≥0.100.

In order to determine whether mAb PD7 was reactive with ribotoxins produced by *A. fumigatus* strains other than isolate Af293, we tested an additional 14 independent isolates of the pathogen recovered from naturally-infested soil samples (Figure 4). We had first established, using ELISA and Western blotting tests of AMM protein precipitates of Af293 (Figure 2), that peak ribotoxin production occurred between 72 h and 96 h post-inoculation. We therefore grew the 14 different isolates (and Af293 as the positive control) in AMM for 72 h, and tested their protein precipitates in the *Afu*-ELISA^®^ and in Western blots using mAb PD7. There were no significant differences in the dry weights of all 15 isolates after 72 h growth in AMM (Figure 4A) and, while detection of the antigen in the ELISA (Figure 4B) was significantly less in a single strain (SX750763) compared to Af293, the absorbance value for this strain was greater than the threshold value for test positivity (≥0.100). Western blots (Figure 4C,D) showed recognition by mAb PD7 of a single immuno-reactive 18 kDa antigen in all 15 isolates, while in the negative control (AMM only) the band was absent (Figure 4C).

We similarly tested other species of *Aspergillus* known to cause invasive pulmonary aspergillosis in humans, namely *Aspergillus niger*, *A. flavus*, *A. nidulans*, and *A. terreus* (Figure S4), and sibling species in the *Aspergillus* section *Fumigati* that have also been reported to cause the disease in humans [40], namely *Aspergillus fumigatiaffinis*, *A. lentulus*, *A. udugawae*, *A. viridinutans*, *Neosartorya fischeri*, and *N. pseudofischeri* (*Aspergillus thermomutatus*) (Figure S5). While the PD7-reactive 18 kDa protein was absent in *A. niger*, *A. flavus*, *A. nidulans*, and *A. terreus* in both *Afu*-ELISA^®^ and Western blotting tests (Figure S4B,C), consistent with the absence of ribotoxins in these species [32], 18 kDa proteins were evident in the culture filtrates of the sibling species, and the positive control *A. fumigatus* Af293 (Figure S5). As with the other species, we initially tested 72-h-old AMM protein precipitates from the sibling species, which showed single PD7-reactive 18 kDa proteins in culture filtrates of *A. lentulus*, *N. pseudofischeri*, *A. viridinutans*, and *N. fischeri*. No PD7-reactive protein was detected in 72-h-old protein precipitates of *A. udugawae* either in *Afu*-ELISA^®^ or Western blotting tests (Figure S5B,C), while protein precipitates of *A. fumigatiaffinis* were positive in the sandwich *Afu*-ELISA^®^ (Figure S5B), and produced two immuno-reactive bands in Western blots, one with a molecular weight of 18 kDa, and a further putative dimer of ~36 kDa (Figure S5C).

To further examine ribotoxin production in these two species, we extended the time period of their culture from 72 to 144 h (Figure S6). Using this extended sampling period, PD7-reactive 18 kDa proteins were detectable in AMM culture filtrates of both species in both the *Afu*-ELISA^®^ (Figure S6B), and in Western blotting tests (Figure S6D,E). The production of the higher molecular weight protein was again evident in 96-h-old culture filtrates of *A. fumigatiaffinis* (Figure S6E), consistent with the production of a dimer by this species [50].

*Afu*-ELISA^®^ and Western blotting studies of protein precipitates from 72-h-old MEB culture filtrates from *Fusarium solani* and *Fusarium oxysporum* (causes of human fusariosis), *Rhizopus oryzae* and *Lichtheimia corymbifera* (causes of human mucormycosis), *Scedosporium aurantiacum* and *Scedosporium apiospermum* (causes of human scedosporiosis), and *Lomentospora prolificans* (the cause of lomentosporiosis) showed the absence of PD7-reactive proteins in these unrelated, but clinically relevant, human pathogenic moulds (Figure S7).

### 3.8. Limit of Detection of the Afu-LFD^®^ Test and Detection of Asp f I in Patient Urine

**Figure 5:**
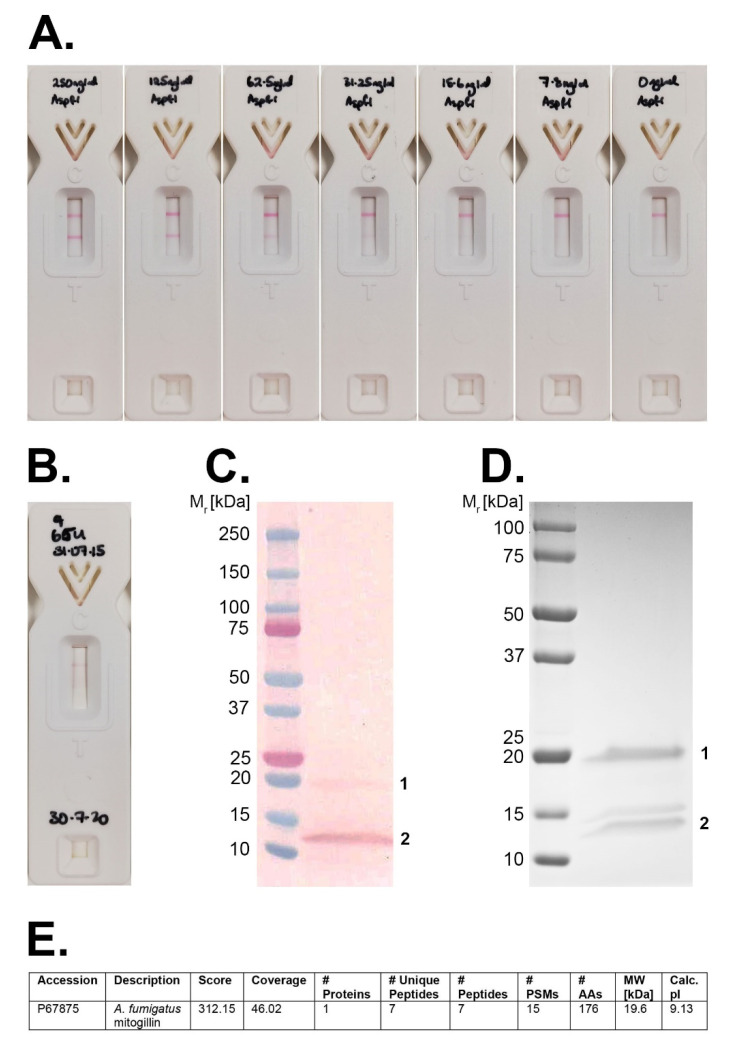
*Afu*-LFD^®^ test results and identification of PD7 immuno-reactive proteins in patient urine sample (**A**). Representative *Afu*-LFD^®^ test results using first volume urine (FVU) from a healthy donor spiked with known concentrations of *A. fumigatus Asp f* I protein. Test results were recorded after 30 min as negative [single internal control (C) line only] or positive [both control (C) and test (T) lines visible] for the PD7 protein biomarker. Strong test lines are visible between 250 ng/mL and 31.25 ng/mL, with faint test lines discernible in samples containing 15.6 ng protein/mL and 7.8 ng protein/mL. No test line is evident in control (un-spiked) FVU (0 ng/mL). The limit of detection of the *Afu*-LFD^®^ test is therefore estimated to be ~15 ng *Asp f* I/mL urine. (**B**). Positive test result [control (C) and test (T) lines present] using a urine sample from a patient with acute lymphoid leukaemia and probable IPA. (**C**) The urine sample was subjected to Western blotting with mAb PD7 and revealed two immuno-reactive proteins with molecular weights of ~19 kDa (labelled 1) and ~12.5 kDa (labelled 2). (**D**) Replica SDS-PAGE gel of the patient urine stained with Coomassie. Bands corresponding to the immuno-reactive proteins in (**C**) were excised and subjected to LC–MS analysis. The ~19 kDa protein (labelled 1) was identified as *A. fumigatus* mitogillin (**E**), synonymous with *A. fumigatus Asp f* I. The identity of the ~12.5 kDa protein (labelled 2) could not be established using this technique.

The limit of detection (LOD) of the *Afu*-LFD^®^ test using FVU samples from healthy donors spiked with the 18 kDa ribotoxin *Asp f* I was ~15 ng protein/mL urine (Figure 5A). Faint test lines were visible using samples containing 15.6 ng protein/mL and 7.8 ng protein/mL, but no test line was visible in negative control urine (un-spiked FVU). Urine from a patient with acute myeloid leukaemia diagnosed with probable IPA according to EORTC guidelines (with microbiological criteria consisting of a serum GM ODI of 3.36 and serum β-D-glucan of 52 pg/mL, followed by a serum GM ODI of 1.84 four days later), yielded a weak but positive *Afu-LFD^®^* test result (Figure 5B). Examination of this sample by Western blotting revealed two PD7-reactive bands with molecular weights of ~19 kDa and ~12.5 kDa (labelled 1 and 2, respectively, in Figure 5C). The corresponding proteins resolved in a replica SDS-PAGE gel stained with Coomassie Blue (Figure 5D) were subjected to LC/MS, which identified the ~19 kDa protein as *A. fumigatus* mitogillin (Figure 5E). The identity of the 12.5 kDa PD7-reactive protein could not be established using this approach.

## 4. Discussion

In this paper, we describe the development and characterisation of an IgG1 monoclonal antibody, PD7, raised against the ribotoxin mitogillin from the human pathogenic mould *Aspergillus fumigatus*, and its use in urine-based diagnosis of pulmonary aspergillosis, the most important invasive mould disease of humans.

The ribotoxins are a family of ribosome-inactivating proteins (RIPs), which display a highly specific ribonucleolytic activity against a single phosphodiester bond in the universally conserved sarcin/ricin domain of 28S ribosomal RNA [51,52], and which have been found to be amongst the most potent and specific inhibitors of eukaryotic translation ever recorded [50,53,54]. The first members of this family (α-sarcin, restrictocin, and mitogillin) were identified as secreted proteins of *Aspergillus giganteus* and *A. restrictus*, and which have potent anti-tumour activity [55]. Subsequently, an 18 kDa IgE-binding allergen from *A. fumigatus*, *Asp f* I, was identified as also being a ribotoxin [33], and was shown to be synonymous with restrictocin, since the original strain of *A. restrictus* used to generate restrictocin had been incorrectly identified and was subsequently re-classified as *A. fumigatus* [56]. Despite this confusion, all *Aspergillus* ribotoxins share a high degree of sequence and structural similarity [52].

In Western blotting studies of recombinant and native ribotoxins, mAb PD7 was found to react with α-sarcin, restrictocin, mitogillin, *Asp f* I, and an 18 kDa antigen secreted by *A. fumigatus* during growth in liquid culture (this study). Recognition of the different ribotoxins from various sources indicate that the mAb binds to an epitope conserved amongst the proteins. To further investigate the ribotoxin-specific nature of the mAb, we developed mutants of the pathogen that lack ribotoxin production. To do this, we used a split marker recombination procedure for targeted gene replacement of the *A. fumigatus* mitogillin-encoding gene (*mitF*) with the hygromycin B phosphotransferase-encoding gene (*hph*). This resulted in hygromycin-resistant mutants that lacked production of the 18 kDa ribotoxin, demonstrated by loss of PD7 binding to this antigen in Western blots of culture filtrates, and in colony immunoblots of the mutant strains. In light of these results, and due to previous ambiguities in *Aspergillus* species identity, we have chosen here to refer to the PD7-reactive ribotoxin produce by *A. fumigatus in vitro* as *Asp f* I.

Previous studies pertaining to ribotoxin generation in *A. restrictus* (*nomen ambiguum*) suggested that production both in liquid and solid culture is associated with conidiophore formation [57,58]. In our studies, we found that *Asp f* I production by *A. fumigatus* in liquid culture occurred following spore germination, an observation consistent with previous investigations [32], and that production ended abruptly at 96 h post-inoculation coincident with hyphal growth cessation. When we compared the dynamics of *Asp f* I production to that of galactofuranose-rich peptidoglycan molecules recognised by the *Aspergillus*-specific mAb JF5 [13,59], we found that it was similarly selectively expressed by the growing fungus only but, unlike Gal*f*-containing peptidoglycans, degraded rapidly once sporulation was initiated. Colony immunoblots of the fungus grown on solid medium showed that ribotoxin production, similar to JF5 antigen production, appeared to be associated with the growing margins, but we were unable to determine using this technique whether this might also be due to the differentiation of immature conidiophores. Consequently, to determine whether *Asp f* I secretion was associated with hyphal proliferation in the absence of sporulation, we investigated its production in a mutant of the pathogen, ΔAf*brlA*, lacking conidiophores [45]. This mutant, created using insertional mutagenesis, lacks a C_2_H_2_ zinc finger transcription factor that activates expression of genes required for asexual development. When grown in liquid culture, the non-sporulating mutant ΔAf*brlA* produced *Asp f* I, with production synchronised to hyphal growth. Taken together, these results show that *Asp f* I production in *A. fumigatus* is secreted during active growth of the pathogen. This is advantageous for pathogen detection during progressive lung infection as it is able to discriminate between invasive hyphal growth and sporulation.

*Aspergillus fumigatus Asp f* I has no orthologs in the genomes of *A. nidulans* and *A. oryzae*, while the occurrence of a gene in *A. terreus* with 41% identity to *Asp f* I, which may be a non-orthologous non-toxin RNAse, has been demonstrated [60]. Using mAb PD7 in ELISA and Western blotting studies, we did not find ribotoxin production in non-*fumigatus Aspergillus* species, consistent with other studies [34,35]. While all species assigned to *Aspergillus* section *Clavati* have been shown to possess ribotoxin-encoding genes [61], the presence of ribotoxin genes in medically-important sibling species within section *Fumigati* [62,63,64,65,66,67,68,69] has only been demonstrated in *Aspergillus viridinutans* and *Aspergillus* (*Neosartorya*) *fischeri* [41]. In ELISA and Western blotting studies using mAb PD7, we demonstrated ribotoxin production in all of the section *Fumigati* species tested. Consequently, mAb PD7 is specific for *A. fumigatus* and clinically-important sibling species. Importantly, the mAb does not cross react with other clinically important mould pathogens. 

The hyphal-specific nature of *Asp f* I production in *A. fumigatus* makes it an ideal candidate for detecting invasive growth of the pathogen. To exploit this property, and previous observations that the 18 kDa ribotoxin, along with other *Aspergillus* biomarkers such as galactomannan [70,71,72,73,74] and the iron siderophore TAFC [75], is a major antigen present in the urine of patients and cattle with IPA [24,25,26], we have used mAb PD7 to develop a lateral-flow assay (*Afu*-LFD^®^) which allows single-step and rapid (30 min) detection of the *Asp f* I biomarker in urine. Unlike Gal*f*-rich peptidoglycans which contain repeat carbohydrate epitopes for detection by mAb JF5 in different sandwich immunoassay formats such as ELISA [10], LFD [59], and proximity ligation assay [76], mAb PD7 binds to a single linear amino acid epitope, which would ordinarily preclude its use in a sandwich immunoassay format. However, the heat-stable nature of the PD7 epitope both in the 18 kDa monomeric ribotoxin *Asp f* I and the 36 kDa dimeric ribotoxin mitogillin, enabled us to incorporate a 10-min heating step at 100 °C that led to aggregation of the 18 kDa species and subsequent detectability by mAb PD7 in the *Afu*-LFD^®^ test. Heating also served to eliminate non-specific binding in the test with normal human urine (results not shown), a phenomenon similarly shown to improve the specificity of the urinary cryptococcal LFA [77]. Heating combined with a urine concentration step resulted in a limit of detection of ~15 ng *Asp f* I/mL urine. The clinical relevance of this LOD has yet to be determined, but we were nevertheless able to detect the antigen in urine from a patient with acute lymphoid leukaemia diagnosed with probable IPA according to EORTC/MSG criteria. However, further large-scale evaluations of the test need to be performed in order to determine its diagnostic efficacy compared to predicate biomarker tests such as the GM ELISA, and during antifungal treatment which is known to reduce the accuracy of antigen-based tests [2]. A limitation of the test is that it only detects *A. fumigatus* and other clinically-relevant species in the section *Fumigati*, but is unable to detect non-*fumigatus Aspergillus* species (*A. flavus*, *A. nidulans*, *A. niger*, and *A. terreus*) which also cause IPA in humans.

In conclusion, the non-invasive nature of the test makes it a potential candidate for diagnosing the disease in patients where recovery of invasive bronchoalveolar lavage fluid is not possible, or where transfer of respiratory fluids to the diagnostic laboratory is undesirable due to the presence of highly contagious pathogens such as the SARS-CoV-2 virus [5]. The *Afu*-LFD^®^ test for *Asp f* I might therefore provide a novel means of identifying *A. fumigatus* lung infections and, when combined with other urine biomarkers [70,71,72,73,74,75], might enable improved and ready detection of this devastating disease. While the presence of *Asp f* I as a circulating biomarker in the bloodstream of infected patients is currently unknown, further studies will aim to determine its presence in serum. Furthermore, its use as a biomarker of *A. fumigatus* lung colonisation in other diseases such as ABPA will also be explored.

## 5. Trademarks

The word marks *Afu*-ELISA^®^ and *Afu*-LFD^®^ [UK00003498611 (granted) and EU018319508/018319499 (pending)] are protected by ISCA Diagnostics Ltd. through the UK Intellectual Property Office (UKIPO) and the European Union Intellectual Property Office (EUIPO).

## Figures and Tables

**Table 1:** Details of fungi used in this study, and summary of *Afu*-ELISA^®^ and *Afu*-LFD^®^ specificities.

Species	Isolate Number	Source ^1^	*Afu*-ELISA^®^	*Afu*-LFD^®^
*Aspergillus flavus*	144B (KP794109)	CRT	-	-
*Aspergillus flavus*	91856iii	IMI	-	-
*Aspergillus fumigatus*	Af293	SK	+	+
*Aspergillus fumigatus*	ΔAf*mitF*::*hph*2.1 ^2^	This study	-	-
*Aspergillus fumigatus*	ΔAf*mitF*::*hph*3.4 ^2^	This study	-	-
*Aspergillus fumigatus*	ΔAf*brl*A7 ^3^	FGSC	+	+
*Aspergillus fumigatus*	Af77	CRT	+	+
*Aspergillus fumigatus*	CM ^4^	CRT	+	+
*Aspergillus fumigatus*	GG ^4^	CRT	+	+
*Aspergillus fumigatus*	SP ^4^	CRT	+	+
*Aspergillus fumigatus*	SX644764 ^4^	CRT	+	+
*Aspergillus fumigatus*	SX705799 ^4^	CRT	+	+
*Aspergillus fumigatus*	SX751776 ^4^	CRT	+	+
*Aspergillus fumigatus*	SX757770 ^4^	CRT	+	+
*Aspergillus fumigatus*	SX787789 ^4^	CRT	+	+
*Aspergillus fumigatus*	SX705795 ^4^	CRT	+	+
*Aspergillus fumigatus*	SX747754 ^4^	CRT	+	+
*Aspergillus fumigatus*	SX746755 ^4^	CRT	+	+
*Aspergillus fumigatus*	SX750763 ^4^	CRT	+	+
*Aspergillus fumigatus*	SX750764 ^4^	CRT	+	+
*Aspergillus fumigatiaffinis*	117186	CBS	+	+
*Aspergillus lentulus*	116882	CBS	+	+
*Aspergillus nidulans*	A4	FGSC	-	-
*Aspergillus niger*	102.40	CBS	-	-
*Aspergillus niger*	1C	CRT	-	-
*Aspergillus niger*	B1	CRT	-	-
*Aspergillus terreus* var. *terreus*	601.65	CBS	-	-
*Aspergillus udugawae*	142231	CBS	+	+
*Aspergillus viridinutans*	121595	CBS	+	+
*Fusarium oxysporum*	167.30	CBS	-	-
*Fusarium solani*	224.34	CBS	-	-
*Lomentospora prolificans*	3.1	CRT	-	-
*Neosartorya fischeri*	544.65	CBS	+	+
*Neosartorya pseudofischeri*	100504	CBS	+	+
*Lichtheimia corymbifera*	T14A (FJ713070)	CRT	-	-
*Rhizopus oryzae*	111233	CBS	-	-
*Scedosporium apiospermum*	117467	CBS	-	-
*Scedosporium aurantiacum*	121926	CBS	-	-

^1^ CBS: Westerdijk Fungal Biodiversity Institute, The Netherlands. SK: S. Krappman, Institute of Clinical Microbiology, Immunology and Hygiene, Universitätsklinikum Erlangen and Freidrich-Alexander-Universität, Germany. FGSC: Fungal Genetics Stock Centre, Kansas City University, USA. CRT: C.R. Thornton, University of Exeter, UK. ^2^ Mitogillin-deficient mutants of *A. fumigatus* Af293 generated in this study. ^3^ Non-sporulating mutant of *A. fumigatus* Af293 [45,46]. ^4^ Strains recovered in this study from naturally-infested soil samples; SX denotes the grid references of peat samples from Dartmoor National Park from which strains were isolated. For *Afu*-ELISA^®^, (+) indicates positive test result, with mean absorbance value greater than the threshold value for test positivity (≥0.100); (-) indicates negative test result, with mean absorbance value less than the threshold value for test positivity. For *Afu*-LFD^®^, (+) indicates positive test result with protein precipitate [both control (C) and test (T) lines visible]; (-) indicates negative test result with protein precipitate [control (C) line visible only].

## Data Availability

The data presented in this study are available on request from the corresponding author. The data are not publicly available due to commercial confidentialities.

## Supplementary Materials

The following are available online at https://www.mdpi.com/2309-608X/7/1/19/s1, Table S1: Details of primers and sequences used for amplification and targeted replacement of the *A. fumigatus* mitogillin-encoding gene *mitF*. Sequences in bold are the reverse complement of the M13R/M13F overlap, Table S2: Kinetic constants of PD7 binding to mitF from *Aspergillus fumigatus* (MBS1189059). Kinetic constants were calculated using ForteBio analysis software, fitting a global 1:1 binding model to binding curves (Figure S1A). A good curve fit to this model was determined as R^2^ > 0.96 and rate constant error <20%. K_ON_ = Association rate constant; K_OFF_ = Dissociation rate constant; K_D_ = Equilibrium dissociation constant (K_ON_/K_OFF_), Figure S1: (A). Binding curves of PD7 to mitF from *Aspergillus fumigatus* (MBS1189059) using Bio-Layer Interferometry (BLI). Association of mitF at 69.44 nM to 277.8 nM to 50 µg/mL of immobilised PD7 for 60 s, followed by dissociation in PBS for 120 s. Background was corrected by subtraction of values from a PD7 bound biosensor, with association and dissociation steps in PBS alone. (B). PTA-ELISA of mAb PD7 against mitF and *Asp f* I. With a threshold absorbance value of 0.100 for test positivity, the limit of detection for both mitF and *Asp f* I was 4 ng/mL, Figure S2: Heat stability the PD7 epitope and protein aggregation. (A). Effect of heat treatment on binding of mAb PD7 to dimeric mitogillin (mitF) or monomeric *Asp f* I in PTA-ELISA. There was no significant effect on mAb binding to mitF over the 60 min period of heat treatment, whereas heating beyond 30 min led to a significant progressive reduction in mAb binding to *Asp f* I. Each bar is the mean of 3 replicates ± SE, and bars with the same letters are not significantly different at *p* < 0.05. (B). Western blot of heat-treated *Asp f* I electrophoresed under native conditions. Heating of the ribotoxin led to a progressive increase in mAb binding indicative of antigen aggregation, Figure S3: Characterisation of ribotoxin-deficient mutants. (A). Agarose gel electrophoresis of PCR products from genomic DNA of the *A. fumigatus* wild type strain Af293 and nine hygromycin-resistant ΔAf*mitF*::*hph* transformants. The products contain both the LF (0.8-kb) and RF (1.0-kb) flanking regions of the *mitF* gene, with either the *mitF* gene (0.5-kb) or assembled *hph* gene (1.6-kb), with predicted sizes of 2.5-kb for strain Af293 or 3.6-kb for gene replacement mutants. (B). Dry weights of Af293 and two independent mutant strains (ΔAf*mitF*::*hph*2.1 and ΔAf*mitF*::*hph*3.4) over the 72-h experimental period. Each data point is the mean of three replicates ±SE. (C) Western blot of pooled AMM protein samples, showing the presence of the PD7-reactive 18 kDa ribotoxin in Af293, but absence in the two independent mutant strains. (D) Western blot of pooled AMM protein precipitates using mAb JF5, showing production of galactofuranose-rich peptidoglycans by all isolates (Af293 and mutants). (E) Colony blots of Af293 and mutant strains processed using mAbs PD7 and JF5. PD7-reactive ribotoxin was detectable at the growing edge of the wild type strain, but was absent in both of the mutant strains. Galactofuranose-rich peptidoglycan production, detected using mAb JF5, was similarly associated with the growing edge of colonies of both Af293 and mutant strains. Scale bar = 0.5 cm, Figure S4: Production of the 18 kDa ribotoxin by non-*fumigatus Aspergillus* species known to cause invasive pulmonary aspergillosis in humans. (A). Dry weights of *A. fumigatus* Af293, three strains of *A. niger*, two strains of *A. flavus*, and a single strain each of *A. nidulans* and *A. terreus*. (B) Absorbance values from *Afu*-ELISA^®^ tests of protein precipitates from 72-h-old AMM cultures of the fungi, showing no detection of ribotoxin protein in the related species. (C) Western blot of pooled protein samples, showing the absence of the PD7-reactive 18 kDa protein in the related species, and in the negative control (AMM only). Bars in (A) and (B) are the means of 3 replicate values ±SE, and bars with the same letter are not significantly different at *p* < 0.05. The threshold absorbance value for detection of ribotoxin protein in the *Afu*-ELISA^®^ (B) is ≥0.100, Figure S5: Production of the 18 kDa ribotoxin by sibling species in the *Aspergillus* Section *Fumigati* known to cause invasive pulmonary aspergillosis in humans. (A). Dry weights of *A. fumigatus* Af293 and sibling species. (B) Absorbance values from *Afu*-ELISA^®^ tests of protein precipitates from 72-h-old AMM cultures of the fungi, showing detection of the protein in *A. lentulus*, *N. pseudofischeri* (*A. thermomutatus*), *A. viridinutans*, *N. fischeri*, and *A. fumigatiaffinis*. (C) Western blot of pooled protein samples, showing the presence of the PD7-reactive 18 kDa ribotoxin in *A. lentulus*, *N. pseudofischeri*, *A. viridinutans*, *N. fischeri*, and *A. fumigatiaffinis*. No ribotoxin was detected in *A. udugawae*, but an additional higher molecular weight band, a putative dimer of ~36 kDa, was evident in *A. fumigatiaffinis*. Bars in (A) and (B) are the means of 3 replicate values ± SE, and bars with the same letter are not significantly different at *p* < 0.05. The threshold absorbance value for detection of antigen in the *Afu*-ELISA^®^ (B) is ≥0.100, Figure S6: Ribotoxin production during extended growth of the sibling species *Aspergillus udagawae* and *Aspergillus fumigatiaffinis*. (A) Dry weights of sibling strains and the positive control strain *A. fumigatus* Af293. (B) Absorbance values for *Afu*-ELISA^®^ tests of protein precipitates of AMM culture filtrates for the three strains at the different time points. (C) Western blot of pooled AMM protein precipitates for Af293 at each time point. (D) Western blot of pooled AMM protein precipitates for *A. udagawae* at each time point (E) Western blot of pooled AMM protein precipitates for *A. fumigatiaffinis* at each time point. Note the presence of single PD7-reactive 18 kDa proteins in all three species, and the presence of an additional higher molecular weight PD7-reactive protein (a putative ~36 kDa dimer) in the pooled 96-h-old samples from *A. fumigatiaffinis*. Data points in (A) and (B) are the means of 3 replicate values ±Se, Figure S7: Production of the 18 kDa ribotoxin by unrelated but clinically important moulds pathogenic to humans. (A). Dry weights of *A. fumigatus* Af293 and the unrelated pathogens. (B). Absorbance values from *Afu*-ELISA^®^ tests of protein precipitates from 72-h-old MEB cultures of the fungi, showing no detection of the 18 kDa protein in the unrelated species. (C) Western blot of pooled protein samples, showing the absence of the PD7-reactive 18 kDa antigen in the unrelated species, and in the negative control (MEB only). Bars in (A) and (B) are the means of 3 replicate values ± SE, and bars with the same letter are not significantly different at *p* < 0.05. The threshold absorbance value for detection of protein in the *Afu*-ELISA^®^ (B) is ≥0.100.
