# Supplementary material for: Aspergillus fumigatus and Its Allergenic Ribotoxin Asp f I: Old Enemies but New Opportunities for Urine-Based Detection of Invasive Pulmonary Aspergillosis Using Lateral-Flow Technology
Source: J Fungi (Basel). 2020 Dec 31;7(1):19. doi: 10.3390/jof7010019 (PMC7823411; doi:10.3390/jof7010019)
Supplement: Supplementary file 1 [file jof-07-00019-s001.pdf]

Table S1. Details of primers and sequences used for amplification and targeted replacement of the *A. fumigatus* mitogillin-encoding gene *mitF*. Sequences in bold are the reverse complement of the M13R/M13F overlap.

Product	Primer	Sequence 5'→3'
LF	mitF_LF.1_F	GAGGCTCGTCTGTAGGTTGA
	mitF_LF.1_R	**GTCGTGACTGGGAAAACCCTGGCG**ATGCGTCTTTTGTCCTCTGC
RF	mitF_RF.1_F	**TCCTGTGTGAAATTGTTATCCGCT**TCTTCGACCACAATATGCGC
	mitF_RF.1_R	ACGAACTAGATTGGCAGCCA
HY	HY split	GGATGCCTCCGCTCGAAGTA
	M13F	CGCCAGGGTTTTCCCAGTCACGAC
YG	YG split	CGTTGCAAGACCTGCCTGAA
	M13R	AGCGGATAACAATTTCACACAGGA
LFHY	mitF_LF.1_F	GAGGCTCGTCTGTAGGTTGA
	HY split	GGATGCCTCCGCTCGAAGTA
RFYG	mitF_RF.1_R	ACGAACTAGATTGGCAGCCA
	YG split	CGTTGCAAGACCTGCCTGAA
Size difference PCR	mitF_LF.1_F	GAGGCTCGTCTGTAGGTTGA
	mitF_RF.1_R	**GTCGTGACTGGGAAAACCCTGGCG**ATGCGTCTTTTGTCCTCTGC

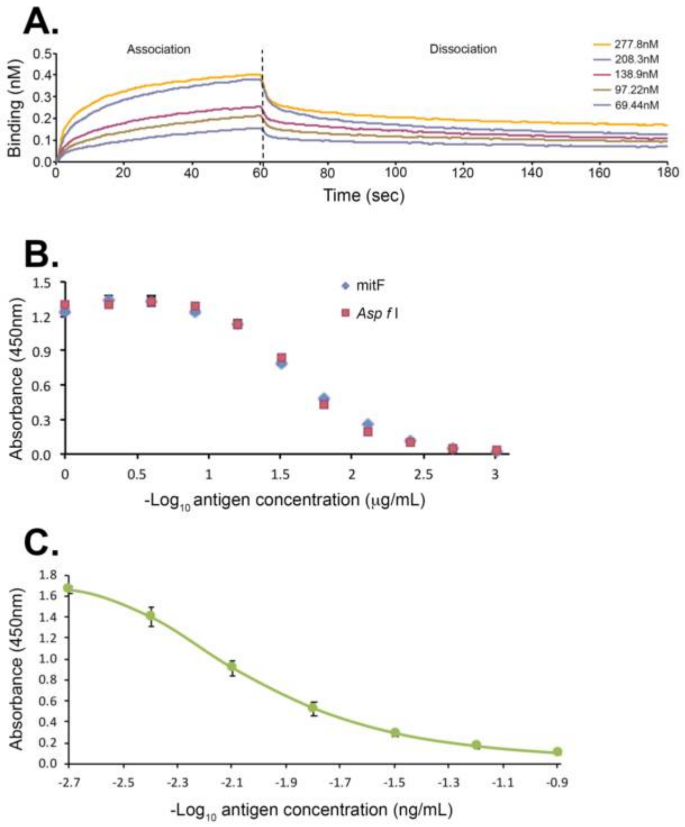

Figure S1. (A). Binding curves of PD7 to mitF from *Aspergillus fumigatus* (MBS1189059) using Bio-Layer Interferometry (BLI). Association of mitF at 69.44 nM to 277.8 nM to 50 μg/mL of immobilised PD7 for 60 s, followed by dissociation in PBS for 120 s. Background was corrected by subtraction of values from a PD7 bound biosensor, with association and dissociation steps in PBS alone. (B). PTA-ELISA of mAb PD7 against mitF and *Asp f* I. With a threshold absorbance value of 0.100 for test positivity, the limit of detection for both mitF and *Asp f* I was 4 ng/mL.

Table S2. Kinetic constants of PD7 binding to mitF from *Aspergillus fumigatus* (MBS1189059). Kinetic constants were calculated using ForteBio analysis software, fitting a global 1:1 binding model to binding curves (Fig. S1A). A good curve fit to this model was determined as R^2 >0.96 and rate constant error <20%. K_{ON} = Association rate constant; K_{OFF} = Dissociation rate constant; K_D = Equilibrium dissociation constant (K_{ON}/K_{OFF}).

mAb	K_{ON} (1/Ms)	K_{OFF} (1/s)	K_D
PD7	$3.11 \times 10^5 \pm 0.24$	$3.18 \times 10^{-2} \pm 0.12$	1.02×10^{-7}

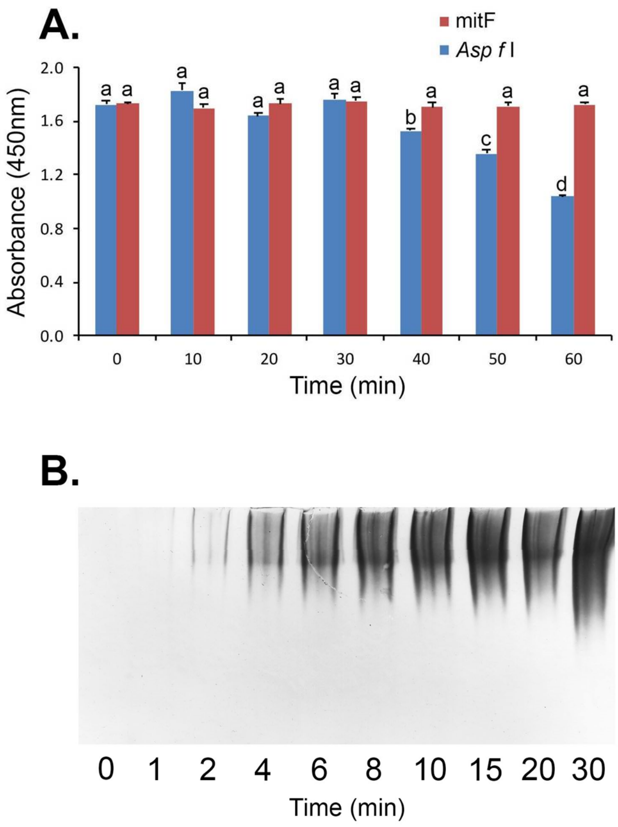

Figure S2. Heat stability the PD7 epitope and protein aggregation. (A). Effect of heat treatment on binding of mAb PD7 to dimeric mitogillin (mitF) or monomeric *Asp f* I in PTA-ELISA. There was no significant effect on mAb binding to mitF over the 60 min period of heat treatment, whereas heating beyond 30 min led to a significant progressive reduction in mAb binding to *Asp f* I. Each bar is the mean of 3 replicates ± SE, and bars with the same letters are not significantly different at $p<0.05$. (B). Western blot of heat-treated *Asp f* I electrophoresed under native conditions. Heating of the ribotoxin led to a progressive increase in mAb binding indicative of antigen aggregation.

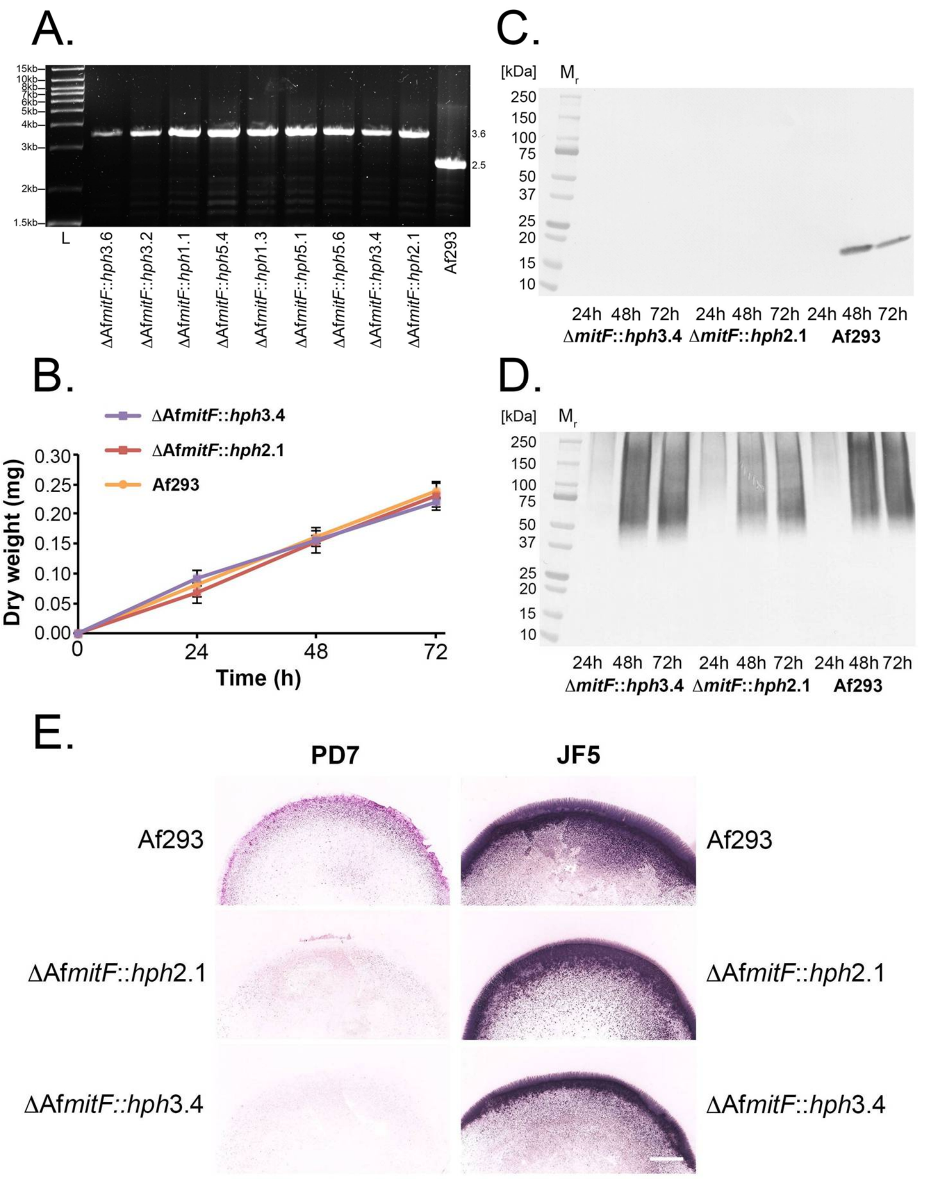

Figure S3. Characterisation of ribotoxin-deficient mutants. (A). Agarose gel electrophoresis of PCR products from genomic DNA of the *A. fumigatus* wild type strain Af293 and nine hygromycin-resistant ΔAf*mitF*::*hph* transformants. The products contain both the LF (0.8-kb) and RF (1.0-kb) flanking regions of the *mitF* gene, with either the *mitF* gene (0.5-kb) or assembled *hph* gene (1.6-kb), with predicted sizes of 2.5-kb for strain Af293 or 3.6-kb for gene replacement mutants. (B). Dry weights of Af293 and two independent mutant strains (ΔAf*mitF*::*hph*2.1 and ΔAf*mitF*::*hph*3.4) over the 72-h experimental period. Each data point is the mean of three replicates ±SE. (C) Western blot of pooled AMM protein samples, showing the presence of the PD7-reactive 18 kDa ribotoxin in Af293, but absence in the two independent mutant strains. (D) Western blot of pooled AMM protein precipitates using mAb JF5, showing production of galactofuranose-rich peptidoglycans by all isolates (Af293 and mutants). (E) Colony blots of Af293 and mutant strains processed using mAbs PD7 and JF5. PD7-reactive ribotoxin was detectable at the growing edge of the wild type strain, but was absent in both of the mutant strains. Galactofuranose-rich peptidoglycan production, detected using mAb JF5, was similarly associated with the growing edge of colonies of both Af293 and mutant strains. Scale bar = 0.5 cm.

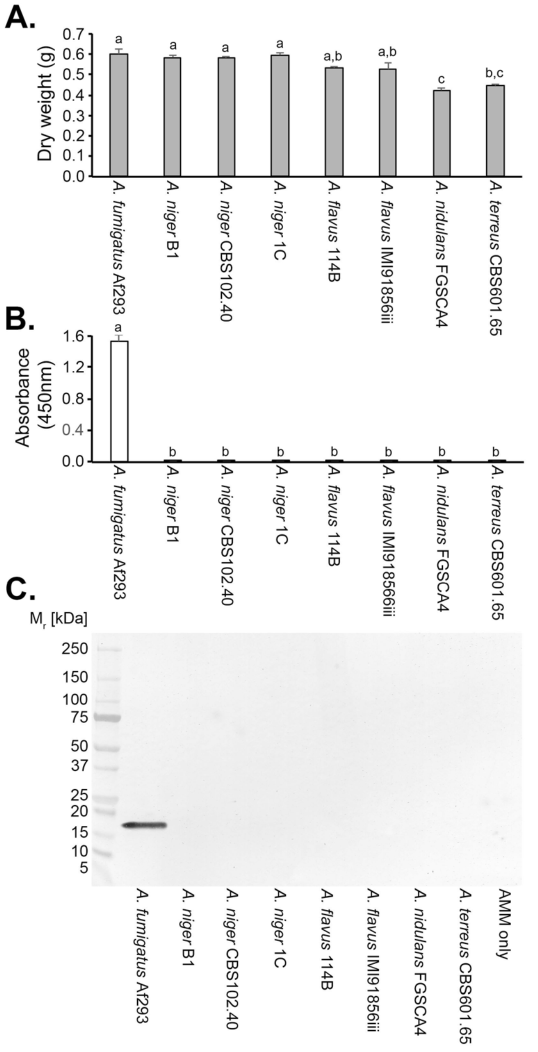

Figure S4. Production of the 18 kDa ribotoxin by non-*fumigatus Aspergillus* species known to cause invasive pulmonary aspergillosis in humans. (A). Dry weights of *A. fumigatus* Af293, three strains of *A. niger*, two strains of *A. flavus*, and a single strain each of *A. nidulans* and *A. terreus*. (B) Absorbance values from *Afu*-ELISA® tests of protein precipitates from 72-h-old AMM cultures of the fungi, showing no detection of ribotoxin protein in the related species. (C) Western blot of pooled protein samples, showing the absence of the PD7-reactive 18 kDa protein in the related species, and in the negative control (AMM only). Bars in (A) and (B) are the means of 3 replicate values ±SE, and bars with the same letter are not significantly different at $p<0.05$. The threshold absorbance value for detection of ribotoxin protein in the *Afu*-ELISA® (B) is ≥0.100.

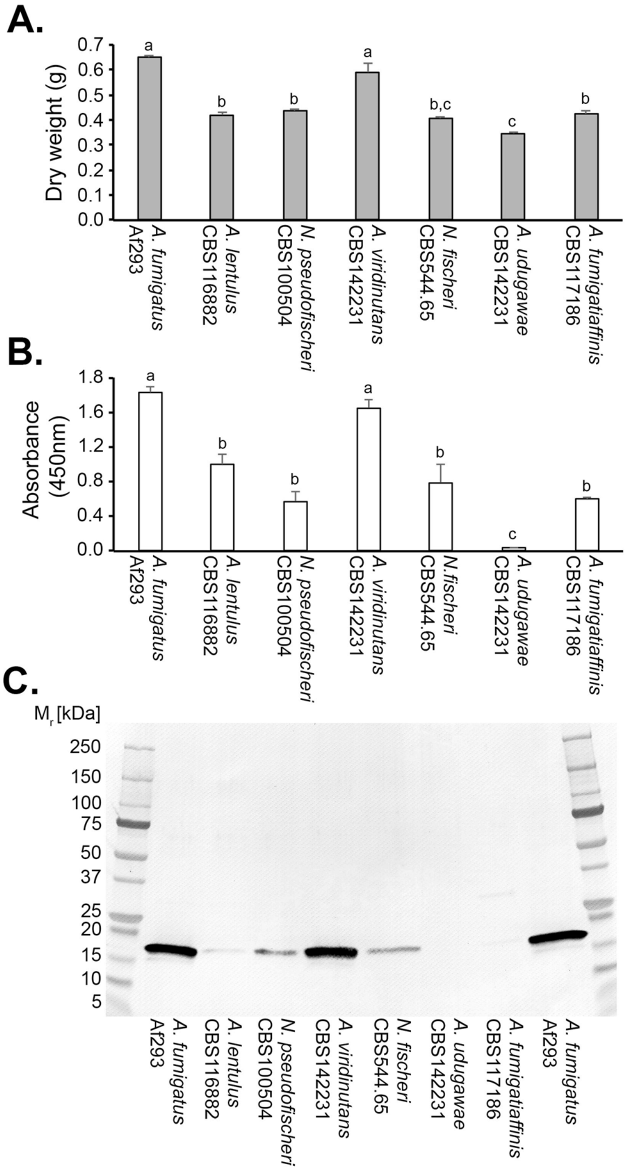

Figure S5. Production of the 18 kDa ribotoxin by sibling species in the *Aspergillus* Section *Fumigati* known to cause invasive pulmonary aspergillosis in humans. (A). Dry weights of *A. fumigatus* Af293 and sibling species. (B) Absorbance values from *Afu*-ELISA® tests of protein precipitates from 72-h-old AMM cultures of the fungi, showing detection of the protein in *A. lentulus*, *N. pseudofischeri* (*A. thermomutatus*), *A. viridinutans*, *N. fischeri*, and *A. fumigatiaffinis*. (C) Western blot of pooled protein samples, showing the presence of the PD7-reactive 18 kDa ribotoxin in *A. lentulus*, *N. pseudofischeri*, *A. viridinutans*, *N. fischeri*, and *A. fumigatiaffinis*. No ribotoxin was detected in *A. udugawae*, but an additional higher molecular weight band, a putative dimer of ~36 kDa, was evident in *A. fumigatiaffinis*. Bars in (A) and (B) are the means of 3 replicate values ±SE, and bars with the same letter are not significantly different at $p<0.05$. The threshold absorbance value for detection of antigen in the *Afu*-ELISA® (B) is ≥0.100.

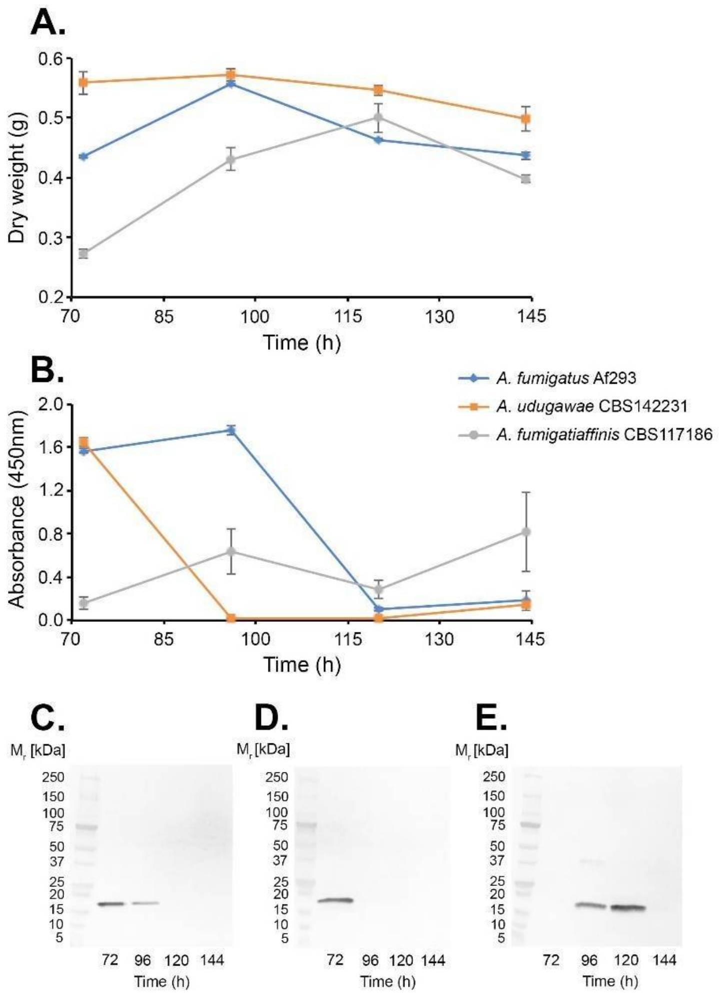

Figure S6. Ribotoxin production during extended growth of the sibling species *Aspergillus udagawae* and *Aspergillus fumigatiaffinis*. (A) Dry weights of sibling strains and the positive control strain *A. fumigatus* Af293. (B) Absorbance values for *Afu*-ELISA® tests of protein precipitates of AMM culture filtrates for the three strains at the different time points. (C) Western blot of pooled AMM protein precipitates for Af293 at each time point. (D) Western blot of pooled AMM protein precipitates for *A. udagawae* at each time point (E) Western blot of pooled AMM protein precipitates for *A. fumigatiaffinis* at each time point. Note the presence of single PD7-reactive 18 kDa proteins in all three species, and the presence of an additional higher molecular weight PD7-reactive protein (a putative ~36 kDa dimer) in the pooled 96-h-old samples from *A. fumigatiaffinis*. Data points in (A) and (B) are the means of 3 replicate values ±SE.

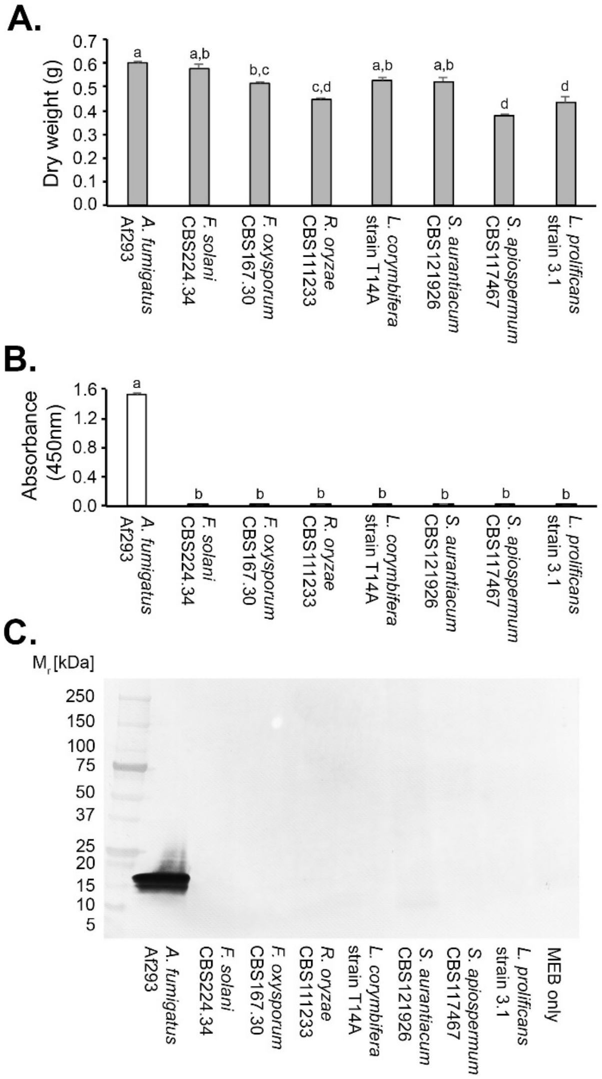

Figure S7. Production of the 18 kDa ribotoxin by unrelated but clinically important moulds pathogenic to humans. (A). Dry weights of *A. fumigatus* Af293 and the unrelated pathogens. (B). Absorbance values from *Afu*-ELISA® tests of protein precipitates from 72-h-old MEB cultures of the fungi, showing no detection of the 18 kDa protein in the unrelated species. (C) Western blot of pooled protein samples, showing the absence of the PD7-reactive 18 kDa antigen in the unrelated species, and in the negative control (MEB only). Bars in (A) and (B) are the means of 3 replicate values ±SE, and bars with the same letter are not significantly different at $p<0.05$. The threshold absorbance value for detection of protein in the *Afu*-ELISA® (B) is ≥0.100.
